# Emerging Technologies and Advanced Strategies in Hemoglobin Defect Screening

**DOI:** 10.3390/jcm14165690

**Published:** 2025-08-12

**Authors:** Cindy Zhang, Victoria Crystal Chen, Bremansu Osa-Andrews, Jing Cao

**Affiliations:** 1 School of Engineering and Computer Science, University of Texas Dallas, Richardson, TX 75080, USA; 2 The Nutritional Sciences and Toxicology Department, University of California Berkeley, Berkeley, CA 94720, USA; 3 College of Medicine, University of Florida, Gainesville, FL 32611, USA; 4 Department of Pathology, University of Texas Southwestern Medical Center, Dallas, TX 75390, USA

**Keywords:** hemoglobin defects, anemia, point of care testing, sickle cell disease, thalassemia

## Abstract

Hemoglobin (Hb) defects, or hemoglobinopathies such as thalassemia and structural Hb variants, are among the most prevalent inherited diseases and are associated with significant mortality and morbidity worldwide. Screening for hemoglobinopathies in the primary care setting plays a critical role in enhancing patient outcomes and advancing population health. It promotes awareness, enables early diagnosis and treatment, supports informed reproductive decisions through genetic counseling, and facilitates access to novel therapies such as genetic modifications. Screening approaches for hemoglobinopathies have evolved to reflect regional prevalence, healthcare infrastructure, and ethical considerations. Varying strategies underscore the necessity of tailoring to local contexts, balancing cost, accuracy, accessibility, and social impact. As global migration reshapes population genetics, flexible and equitable screening frameworks are increasingly essential. This review focuses on practical techniques suitable for the screening of Hb defects in primary care. Recent advances and findings in high-performance liquid chromatography, capillary zone electrophoresis, mass spectrometry, point of care testing, and molecular methodologies are covered. In addition, strategies and approaches in multiple regions in the world are reviewed.

## 1. Introduction

Hemoglobin (Hb) defects are among the most common inherited defects in humans. A report from the World Health Organization (WHO) in 2008 revealed that about 5.2% of the world’s population have a significant genomic variant that results in Hb defects, and that 24% are carriers of a known variant [1]. It has deep implications for life quality from lifetime medication, transfusion, reproductivity risk to mild symptoms like anemia that are refractory to nutrient supplementation [2]. Therefore, there is a great demand for screening and diagnosis of Hb defects. Correspondingly, there is an array of testing methodologies available and testing strategies utilizing one or multiple methods.

Screening for Hb defects has seen significant global expansion in recent years. This trend reflects the need for screens due to the severe impact of these defects and the availability of increasingly accurate and cost-effective testing tools [3,4]. This review will discuss 1. Types of Hb defects; 2. Methodologies for the evaluation of Hb defects; 3. Recent advances in population screen strategies. Ultimately, this article elaborates on the selection of solutions based on different needs in population health.

## 2. Types of Hb Defects

Hb is a tetrameric protein consisting of two alpha-like (α-like) and two beta-like (β-like) globin chains, each bound to an iron-containing heme group, enabling oxygen transport to tissues and organs. Adult Hb A is composed of two α and two β globin chains (α2β2) and constitutes 95–98% of total Hb in healthy adults from 6 to 8 months of age. Fetal Hb (Hb F), composed of two α and two γ globin chains (α2γ2), is predominant during fetal development but declines to less than 1% by two years of age [5]. Hb A2 is composed of two α and two δ globin chains (α2δ2) and constitutes 2.5–3.5% of total Hb in adults. Throughout the life of an individual without Hb defects, the production of α-like and β-like chains remains balanced, ensuring no excess of either. Most Hb defects result from missense mutations in globin gene coding sequences, while copy number variations (e.g., deletions, duplications) and mutations in regulatory regions affecting transcription and RNA stability also contribute. There have been over 1000 specific mutations reported that result in inherited Hb defects, classified into qualitative defects (structural Hb variants) and quantitative defects (thalassemias) [6].

### 2.1. Qualitative Hb Defects

Structural Hb variants may result in changes to the Hb molecule’s primary, secondary, tertiary, or quaternary structure and often affect their functions, leading to conditions like sickle cell disease (SCD). Hemolytic SCD encompasses several distinct genotypes, including homozygous HbSS, a single base-pair point mutation (GAG to GTG) results in the replacement of the amino acid glutamic acid (Glu, hydrophilic) by valine (Val, hydrophobic), compound heterozygous conditions including HbSC, HbSOArab, HbSD, HbSE, and HbS in combination with beta-thalassemia major or thalassemia intermedia. SCD is particularly prevalent in populations from sub-Saharan Africa, the Caribbean, Saudi Arabia, India, and the Mediterranean regions of Greece and Italy [7,8].

The major functional consequences of Hb structural variants include: 1. Changes in solubility: Common mutations in beta globin genes, such as Hb S and Hb C, alter the solubility of hemoglobin. Hb S polymerizes when deoxygenated, while Hb C forms crystals; 2. Hb instability: Unstable Hb variants precipitate under stress, causing hemolytic anemia. Denatured hemoglobin forms Heinz bodies in red blood cells, which can damage the cell membrane; 3. Altered oxygen affinity: Variants can increase or decrease Hb’s affinity for oxygen, causing polycythemia or anemia. Variants like Hb Chesapeake increase oxygen affinity, causing erythrocytosis, while low-affinity variants, such as Hb Kansas, cause cyanosis and anemia; 4. Heme oxidation: Mutations affecting the heme binding site can produce methemoglobin due to oxidized iron in heme [6,9].

Heterozygous hemoglobin traits are often not associated with serious symptoms in childhood, for which reason they are usually identified only in adulthood. Hemoglobin S trait (Hb AS), hemoglobin E trait (Hb AE), and hemoglobin C trait (Hb AC) are among the commonest mild or asymptomatic hemoglobin traits. While these do not cause serious disease, the presence of at least one abnormal hemoglobin may negatively influence a test on a complete blood count (CBC) panel, enough to warrant a hemoglobinopathy screening, which may eventually lead to the identification of the variant genotype. Hemoglobin E, the most common variant globally, affecting more Asians, is known to cause microcytosis due to reduced synthesis of the beta-globin chain linked to unstable mRNA [10]. More importantly, an individual with a carrier or non-sickling variants like C and D may give birth to a sickling offspring if the spouse carries the S variant.

### 2.2. Quantitative Hb Defects

While structural variants alter Hb’s structure and function, thalassemias lead to a reduction in the amount of globin chain production, causing an altered α-like to β-like chain ratio. This imbalance leads to excess chains aggregating, causing premature destruction of RBCs. β thalassemias are caused by mutations in regulatory elements of the β globin gene and are classified as β+ (minor) if some β globins are produced or β^0^ (major) if none is produced. Excess α chains aggregate, damaging RBC membranes and leading to ineffective erythropoiesis. α thalassemias result from deletions of one or more α globin genes (HBA1 and HBA2) on chromosome 16 and are classified based on the number of deleted or mutated genes. Conditions range from silent carriers (one gene deleted) to Hb H disease (three genes deleted) and hydrops fetalis (four genes deleted) [6].

### 2.3. Γ, Δ Globin Mutations, and Combined Effects

Mutations in γ and δ globin genes cause a range of defects, from benign hereditary persistence of fetal Hb, HPFH, a benign condition, to δβ thalassemia that is symptomatic. Some mutations result in both qualitative and quantitative defects. For example, Hb Lepore results from a fusion of δ- and β-globin genes with low globin expression [11].

## 3. Testing Methodologies

Methods for analyzing Hbs can be broadly divided into protein-based (protein chemistry) and DNA-based (molecular genetic) methods. In this methodology session, we will mainly cover high-throughput protein-based methods that fit the needs of population screening, including high-performance liquid chromatography (HPLC), capillary zone electrophoresis (CZE), mass spectrometry (MS), and more recent approaches of point of care testing (POCT).

Family history and hematology tests—such as CBC, reticulocyte count, and blood smear—combined with protein-based Hb analysis are often sufficient to diagnose common Hb variants. Thalassemia screening also begins with protein-based methods, which may reveal abnormal Hb ratios (e.g., elevated Hb A2 or sometimes Hb F), but confirmation, especially for α-thalassemia, requires DNA testing [8].

Historically, gel-based electrophoresis methods were used to evaluate many common hemoglobinopathies. Electrophoresis can resolve many normal and abnormal Hbs, such as Hb A, Hb A2, Hb F, Hb S, and Hb C. Several gel electrophoresis techniques have been routinely used in clinical laboratories, including acid or alkaline gel electrophoresis and isoelectric focusing (IEF).

### 3.1. Alkaline and Acidic Gel-Based Electrophoresis

Electrophoresis separates Hbs based on charge by applying an electric field to a gel at either alkaline (cellulose acetate) or acidic (citrate agar) pH. It can detect and semi-quantify Hb A, A2, F, and variants like Hb S and C, making it a low-cost, widely used method for screening common Hb disorders. However, limitations include co-migration of some Hbs—such as Hb A2, C, E, and O at alkaline pH—and difficulty distinguishing fast-moving variants (e.g., Hb H, Hb Barts) or quantifying low-abundance Hbs like Hb A2. Acidic pH helps separate some co-migrating variants, but still cannot resolve others like Hb E from A2 or Hb D from G. [12].

### 3.2. IEF

IEF separates Hbs based on their net charge within a pH gradient that sets the isoelectric point (pI]) on a gel medium (e.g., polyacrylamide, thin-layer agarose). The Hb migration pattern of IEF is similar to alkaline gel electrophoresis, but with resolution of Hb C from Hb E and Hb O, and Hb S from Hb D and Hb G. In addition, Hb A and Hb F are clearly resolved. IEF can detect fast-migrating Hbs like Hb H and Hb Barts, making it useful for newborn screening (NBS). However, it cannot distinguish Hb E from Hb C-Harlem or Hb O-Arab, nor Hb G-Philadelphia from Hb Lepore [8]. It also lacks precision for quantifying low-abundance Hbs like Hb A2 and, like gel electrophoresis, is unsuitable for high-throughput use due to manual processing.

### 3.3. HPLC

In ion-exchange HPLC, Hbs bind to a negatively charged resin and are eluted by a positively charged buffer in increasing concentration. Elution rates depend on each Hb’s affinity for the column and are detected by a photometer. Peaks are displayed in defined “windows” based on retention time, which varies by instrument and software. Hb fractions are quantified by calculating the area under each peak [13]. Cation-exchange HPLC separates many normal and variant Hbs—including A, A2, F, S, C, O-Arab, D-Punjab, and G-Philadelphia—allowing broader detection than gel electrophoresis. It is widely used in population screening, including prenatal and NBS [14], especially in areas with high prevalence of SCD and β-thalassemia [15]. HPLC is also useful for detecting β-thalassemia carriers and for quantifying Hb A2 and F in individuals undergoing transfusion or hydroxyurea therapy [16].

Limitations include overlap of certain Hbs (e.g., E, Korle-Bu, Lepore) with Hb A2, which affects quantification. Hb A2 may also be falsely elevated with Hb S. Instrument-specific separation differences, post-translationally modified Hbs, and artifacts can complicate interpretation, requiring expert review [17,18].

### 3.4. CZE

CZE separates Hbs in a liquid buffer using electro-osmotic flow through negatively charged silica capillaries. The output is a tracing with quantifiable peaks representing Hb variants. It can be used with dried blood spots, cord blood, or whole blood, making it applicable to NBS [19]. CZE offers resolution and throughput comparable to HPLC, with automation and low sample volume. It is not affected by glycated or acetylated Hbs and clearly separates Hb E from A2, allowing accurate quantification. Drawbacks include higher cost, need for skilled personnel, and limited separation of Hb S from D [20,21].

**Figure 1:**
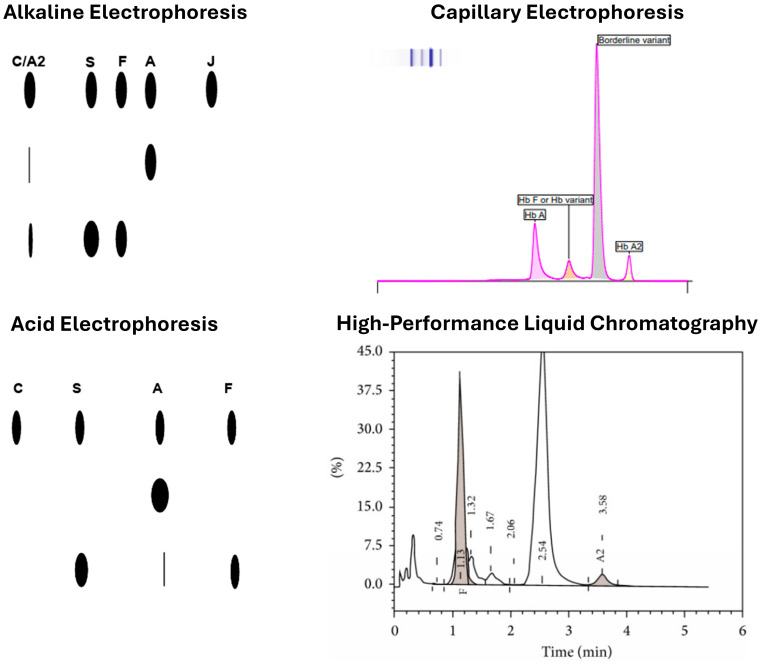
Examples of hemoglobinopathy testing results from alkaline and acid gel, capillary zone electrophoresis and high-performance liquid chromatography.

Both HPLC and CZE offer high resolution, reproducibility, small sample requirement, automation, and fast turnaround, supporting their widespread use [21]. They are often complementary: one may resolve variants, but the other cannot. Notably, recent transfusion (>3 months) can affect all protein-based methods by masking the patient’s native Hb profile. Examples of results from gel-based methods and high-throughput methods are shown in Figure 1.

### 3.5. MS

Matrix-Assisted Laser Desorption/Ionization Time-of-Flight (MALDI-TOF) MS has been investigated as a transformative, cost-effective, high-throughput, and accurate screening method for the utility of population Hb defect screen. Zhang et al. developed a machine learning model for quantifying Hb chains to screen thalassemia [22]. This novel method was tested on 674 samples from asymptomatic carriers, symptomatic patients, and controls. By analyzing hemoglobin imbalance parameters (e.g., α/β and α-β ratios), logistic regression models were constructed and validated. The model achieved an ROC-AUC of 0.99 with 98.7% sensitivity and 95.5% specificity for general thalassemia detection. A separate model for β-thalassemia identification demonstrated strong performance (AUC 0.94, sensitivity 96.5%, specificity 87.8%). The study highlights this approach’s potential for rapid, affordable screening in large populations. Another study extends MALDI-TOF MS applications to β-globin variant and β-thalassemia screening and classification [23]. Using 901 training samples with known genotypes, a model was developed to analyze ratios of δ- to β-globin, γ- to α-globin, and γ- to β-globin, alongside detecting abnormal globin-chain patterns. The method was validated in 16,172 participants and 201 β-thalassemia patients. It achieved high accuracy (99.78%) and specificity (99.89%), outperforming traditional methods (CBC and CZE) in sensitivity (97.52% vs. 88.01%). The approach identified novel abnormal Hbs and classified thalassemia major and thalassemia intermedia cases with high reliability, correctly classifying 178 out of 201 patients.

Together, these studies establish MALDI-TOF MS as a potential game-changing technology for Hb defect screen. The integration of machine learning and direct globin-chain measurement enables precise and efficient screening, paving the way for widespread clinical implementation.

Diagnosis of Hb defects using liquid chromatography tandem mass spectrometry (LC-MS/MS) is used clinically [24]. There is also a report on using CZE coupled with high-resolution orbitrap MS to detect both Hb variants and beta thalassemia [25]. However, these methods lack cost efficiency and throughput to be adopted in Hb defect screening.

### 3.6. POCT

POCT, or near-patient testing, involves performing analytical tests outside traditional laboratories, offering rapid and accessible diagnostics. POCT has gained popularity for Hb defect screening due to its portability, ease of use, low blood volume requirement, and immediate results. This has driven demand for innovative, user-friendly, and cost-effective technologies that enable de-centralized care with minimal sampling, as shown in Table 1. In some developing countries, the Naked Eye Single Tube Red Cell Osmotic Fragility Test (NESTROFT), a cost-effective, rapid and reliable screening test for the detection of β-thalassaemia trait, is largely used as a first approach [26]. The Sickle SCAN assay, a POCT immunoassay, achieved high sensitivity (98.1%) and specificity (91.1%) for SCD diagnosis [27]. Another POCT immunoassay HemoTypeSC had an overall sensitivity of 99.5% and specificity of 99.9% across all hemoglobin phenotypes, SCD, hemoglobin C disease, and the related carrier states [28].

Other examples of POCT Hb screening devices include Gazelle, a microchip electrophoresis-based portable device, which demonstrated 99% accuracy for detecting Hb variants (HbSS, Thalassemia Major, sickle cell trait) in low-resource settings [29]. HemeChip is a miniaturized and paper-based microchip electrophoresis device that identifies the most common hemoglobin variants (Hb S, C, and E) with 100% sensitivity, and an overall diagnostic accuracy of 98.4% [30]. It can further integrate anemia detection and Hb variant identification [31]. The integration of cutting-edge technologies such as microfluidics, nanotechnology, telemedicine, and materials science could overcome current limitations, enabling compact and possibly disposable POCT devices for broader home and bedside use. Table 2 shows the main features and applications of methods for Hb defects screening.

In addition to identifying specific types of Hb defects, since not all Hb variants are harmful, tests of altered Hb oxygen affinity (HOA) may also be used for routine abnormal Hb detection. Patel et al. validated the oxygen dissociation assay, a rapid, cost-effective platform capable of screening and characterizing HOA modifiers with strong correlation to reference methods [32]. Sekyonda et al. presented a rapid, low-complexity optical assay that accurately measures HOA and effectively distinguishes between normal and SCD Hb variants, also enabling assessment of treatment responses in SCD [33].

## 4. Screening Strategies

Screening for Hb defects serves multiple purposes, each aimed at improving patient care and population health. First, it raises awareness, helping individuals understand their condition and potential health implications. Early detection facilitates timely and effective treatment, which can significantly improve outcomes. Screening also supports family planning by allowing for genetic counseling and informed reproductive choices. Furthermore, it enables access to emerging treatments, such as gene therapy, offering new hope for managing hemoglobinopathies. Ultimately, early management of symptoms enhances overall quality of life for affected individuals.

Structural hemoglobin variants, such as SCD and Hb C, are highly prevalent in populations from regions like sub-Saharan Africa, the Caribbean, Saudi Arabia, India, and the Mediterranean [8]. These variants often cause hemolysis and associated complications. Thalassemias are common in Southeast Asia and Mediterranean countries. Large α deletions are common in Southeast Asian populations. Accordingly, a variety of guideline recommendations and practicing strategies for hemoglobinopathies and thalassemia in at-risk individuals are implemented in different regions.

### 4.1. Guidelines on Hb Defect Screening

The WHO advises screening for thalassemia and other Hb defects in all individuals of reproductive age [34,35]. The American College of Obstetricians and Gynecologists (ACOG)’s Committee on Genetics previously recommended that Hb electrophoresis should be performed following a CBC with low mean corpuscular hemoglobin (MCH) concentration or mean corpuscular volume (MCV) or if there is suspicion of hemoglobinopathy based on ethnicity (African, Mediterranean, Middle Eastern, Southeast Asian, or West Indian descent), although it does not dictate which electrophoresis method (CZE, cellulose-acetate electrophoresis or IEF) [36]. In a more recent recommendation, ACOG has expanded the recommendation to offering universal hemoglobinopathy testing to persons planning pregnancy or at the initial prenatal visit if no prior testing results are available for interpretation [37].

Osa-Andrew et al. investigated the selection of CZE as a single method screening in the general population [38]. In a single-center institution, during a 12-month period, 373 adult patients without previously diagnosed Hb variants had tests for Hb defects ordered. The performance of the single CZE method was compared against a comprehensive algorithm, which uses both CZE and HPLC as the first line tests with a reference to more advanced variant identification, such as MS and genetic analyses. The CZE method successfully quantified common Hb fractions and therefore detected Hb SS, S trait, SC, beta-thalassemia, decreased A2, delta chain variants, and elevated Hb F. It also captured the presence of uncommon hemoglobin variants, including Hemoglobin E trait, G-Philadelphia, and Hemoglobin Q-India, to be further tested for identification of the variant. Therefore, CZE was shown to be an effective electrophoresis screening method for Hb defects.

### 4.2. Evolving Screening Approaches

Screening strategies have adapted to regional needs and healthcare infrastructure. In the United States, universal neonatal screening was implemented across all 50 states by 2006, following a formal recommendation by the National Institutes of Health two decades earlier [39]. This universal screening initiative allows for early intervention, including prophylactic penicillin, immunizations, preventive care, education, and disease-modifying therapies, all aimed at reducing morbidity and mortality.

**Figure 2:**
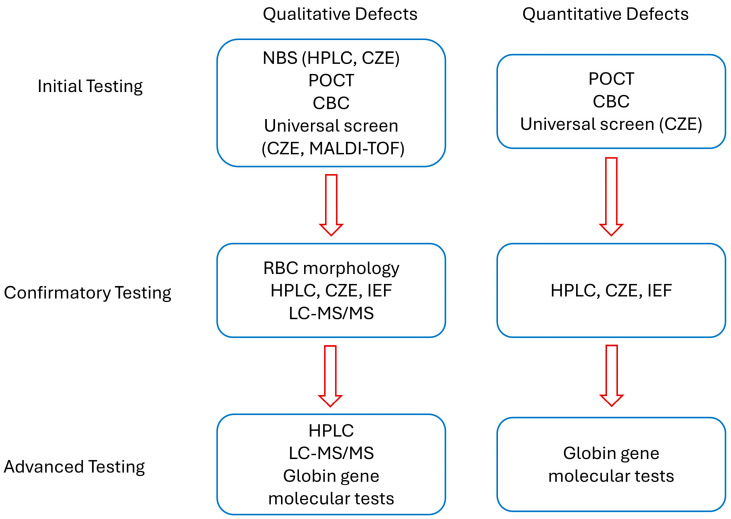
Current common practices for Hb defects screening, confirmation, and advanced follow-up tests.

Similarly, the United Kingdom has implemented universal neonatal screening [40], while other European countries focus on targeted screening for recent immigrants [41]. In Mediterranean and Middle Eastern regions, where thalassaemia is prevalent, prenatal testing programs have been prioritized, although their effectiveness remains inconsistent [42,43]. A summary of current common practices for Hb defects testing is shown in Figure 2.

A recent investigation conducted in Mali—where sickle cell disease (SCD) is highly prevalent but healthcare resources are limited—assessed the practicality of employing rapid diagnostic tests (RDTs) for newborn screening [44]. Researchers examined the performance of two immunoassay-based point-of-care tests to detect hemoglobin variants HbA, HbS, and HbC from more than 4000 cord blood samples obtained during childbirth. The study spanned four months and included multiple hospitals, with maternal consent being a prerequisite for participation. Around 80% of newborns were tested using these RDTs. However, some deliveries occurred too quickly for consent to be obtained, and a small proportion of mothers declined participation, likely due to fears or stigma surrounding the disease. While point-of-care testing facilitated timely results, delivering a diagnosis so soon after birth proved difficult without adequate counseling services. When compared with high-performance liquid chromatography (HPLC), the RDTs showed roughly 80% concordance but often failed to detect beta-plus thalassaemia. Despite these shortcomings, the study supported the viability and cost-effectiveness of RDT-based screening, noting that infants not carrying HbS or HbC could be reliably excluded from further evaluation, while positive cases required confirmatory testing via HPLC [44].

In contrast, Denmark has implemented a prenatal screening model aimed at identifying carrier status among parents from ethnic groups with a haemoglobinopathy prevalence above 1% [45]. Over 16 years, this targeted initiative has tested approximately 25,000 adults using HPLC and genetic analysis, leading to the detection of 61 affected pregnancies. The program involves general practitioners and midwives, who assess ethnic background during preconception consultations or early in pregnancy. This approach prioritizes informed reproductive choices to mitigate the future burden of severe hemoglobin defects. However, it also introduces ethical complexities, including concerns about labeling individuals as carriers, potential stigma, and societal pressures around family planning decisions.

These contrasting models underscore key challenges in designing and implementing haemoglobinopathy screening programs. Mali’s case highlights issues of affordability, test accuracy, and infrastructure when considering the expansion of RDT screening to meet WHO goals for universal newborn screening. Lowering the cost of each RDT—currently between $3 and $10—to under $1 USD could significantly improve scalability in low-income settings. Nonetheless, with a relatively low disease detection rate of 1–2% among neonates, the cost-effectiveness of universal screening must be carefully weighed [4].

Meanwhile, Denmark’s strategy brings forward sensitive questions about social responsibility, reproductive autonomy, and bioethics. One central issue is whether individuals who are carriers or affected by conditions like SCD should be counseled against starting families due to potential genetic risks. While genetic counseling aims to inform and support, it risks crossing into territory where individuals may feel pressured or stigmatized for their reproductive choices, raising concerns about eugenics and reproductive discrimination.

The role of society in culturally sensitive decisions such as pregnancy termination is also ethically fraught. For some families, religious or cultural beliefs may preclude termination, even in cases of severe Hb defects. National screening strategies must therefore balance public health goals with respect for individual autonomy, cultural diversity, and informed consent. Ethical frameworks should ensure that decision-making remains voluntary and non-directive, supported by culturally competent counseling.

Moreover, determining which hemoglobin defects justify intervention further complicates the ethical landscape. For instance, while severe conditions like transfusion-dependent thalassemia may clearly benefit from early detection and potential prevention strategies, the same clarity may not exist for milder or variable conditions. Distinctions between disease severity, treatment burden, and quality of life are not always straightforward and may shift over time with advances in therapy. These decisions must avoid pathologizing benign traits or marginalizing affected individuals and should be guided by transparent criteria developed through inclusive, multidisciplinary dialog.

Together, these findings emphasize the need for context-specific screening frameworks. Universal newborn testing may be more appropriate for high-burden countries like Mali, whereas targeted screening remains effective in low-prevalence settings such as Denmark. As international migration shifts demographic landscapes, it becomes increasingly important to develop fair, efficient, and ethically sound screening policies. Technological innovations, alongside culturally sensitive planning, will be critical in ensuring that these programs yield reliable and meaningful health outcomes.

## 5. Conclusions

Advancements in the methods for screening and diagnosing hemoglobinopathies have significantly improved. Hemoglobin electrophoresis, a robust and straightforward technique, remains widely used. Traditional methods like cellulose acetate and acid citrate gel electrophoresis are now largely replaced by IEF, which allows for simultaneous testing of multiple samples. High-accuracy methods such as CE, HPLC, and LC-MS/MS provide precise results but are often unsuitable for low-resource settings due to high costs and technical requirements. MALDI-TOF MS and molecular genetic testing in large populations demonstrate the potential to implement universal screening in compliance with recommendations from professional societies. RDT realized through POCT has emerged as an alternative in the past decade. These methods include lateral flow immunoassays and single-use electrophoresis kits, which are more accessible and user-friendly. Despite their limitations, RDTs provide an immediate and practical option, particularly in resource-limited environments. The selection of screening approaches requires tailoring according to local resources, budget, social, and ethnic considerations.

## Figures and Tables

**Table 1:** Performance and features of POCT devices for Hb defect screening.

Device Name	Technology	Target Conditions	Sensitivity	Specificity	Notable Features
NESTROFT	Naked Eye Osmotic Fragility Test	β-thalassemia trait	Not specified	Not specified	Cost-effective, rapid, reliable; used as initial screen in developing countries
Sickle SCAN	Immunoassay POCT	SCD	98.10%	91.10%	Portable, rapid test for SCD diagnosis
HemoTypeSC	Immunoassay POCT	SCD, hemoglobin C disease, and carrier states	99.50%	99.90%	High accuracy across various hemoglobin phenotypes
Gazelle	Microchip electrophoresis	HbSS, Thalassemia Major, sickle cell trait	99% accuracy	Not specified	Portable, suitable for low-resource settings
HemeChip	Paper-based microchip electrophoresis	Hb S, C, and E variants	100%	Not specified	Diagnostic accuracy: 98.4%; also detects anemia; miniaturized, paper-based format

**Table 2:** Features and applications of methods for Hb defects screening.

Method	Cost per Test	Accuracy/Resolution	Throughput	Applicability/Settings
HPLC	Moderate	High (good separation of Hb variants, including HbA2 and HbF)	High (automated analyzers allow batch processing)	Widely used in reference and hospital labs; standard in newborn and carrier screening programs
CZE	Moderate	High (better resolution for some variants than HPLC, e.g., HbE vs. HbA2)	High (similar to HPLC with modern systems)	Suitable for hospital and reference labs; increasingly replacing HPLC in clinical labs
MALDI-TOF	Moderate	Very high (can detect rare and modified hemoglobins, structural variants, and globin chain imbalances)	Moderate (requires specialized workflow)	Best suited for specialized or confirmatory testing centers; not yet standard for population-wide screening
POCT	Low–Moderate	Moderate (can detect major variants like HbS and HbC but not minor ones reliably)	Very high (minimal prep, rapid results)	Ideal for field settings, resource-limited areas, or urgent clinical triage; limited as a standalone screening tool

## Abbreviations

The following abbreviations are used in this manuscript:

ACOG	American College of Obstetricians and Gynecologists
CBC	complete blood count
CZE	capillary zone electrophoresis
Hb	Hemoglobin
Hb F	Fetal Hb
HOA	Hb oxygen affinity
HPLC	high-performance liquid chromatography
IEF	isoelectric focusing
MALDI-TOF	Matrix-Assisted Laser Desorption/Ionization Time-of-Flight
MS	mass spectrometry
NBS	newborn screening
POCT	point of care testing
RDT	rapid diagnostic tests
SCD	sickle cell disease
WHO	World Health Organization
